# The complete genome sequencing of *Prevotella intermedia* strain OMA14 and a subsequent fine-scale, intra-species genomic comparison reveal an unusual amplification of conjugative and mobile transposons and identify a novel *Prevotella-*lineage-specific repeat

**DOI:** 10.1093/dnares/dsv032

**Published:** 2015-12-08

**Authors:** Mariko Naito, Yoshitoshi Ogura, Takehiko Itoh, Mikio Shoji, Masaaki Okamoto, Tetsuya Hayashi, Koji Nakayama

**Affiliations:** 1 Division of Microbiology and Oral Infection, Department of Molecular Microbiology and Immunity, Nagasaki University Graduate School of Biomedical Sciences, Nagasaki 852-8588, Japan; 2 Department of Bacteriology, Faculty of Medical Sciences, Kyushu University, Higashi-ku, Fukuoka 812-8582, Japan; 3 Department of Biological Information, Tokyo Institute of Technology, Meguro-ku, Tokyo 152-8550, Japan; 4 Department of Oral Microbiology, Tsurumi University, School of Dental Medicine, Tsurumi-ku, Yokohama 230-8501, Japan

**Keywords:** *Prevotella intermedia*, complete genome sequence, conjugative transposon, mobilizable transposon, repeat sequence

## Abstract

*Prevotella intermedia* is a pathogenic bacterium involved in periodontal diseases. Here, we present the complete genome sequence of a clinical strain, OMA14, of this bacterium along with the results of comparative genome analysis with strain 17 of the same species whose genome has also been sequenced, but not fully analysed yet. The genomes of both strains consist of two circular chromosomes: the larger chromosomes are similar in size and exhibit a high overall linearity of gene organizations, whereas the smaller chromosomes show a significant size variation and have undergone remarkable genome rearrangements. Unique features of the *Pre. intermedia* genomes are the presence of a remarkable number of essential genes on the second chromosomes and the abundance of conjugative and mobilizable transposons (CTns and MTns). The CTns/MTns are particularly abundant in the second chromosomes, involved in its extensive genome rearrangement, and have introduced a number of strain-specific genes into each strain. We also found a novel 188-bp repeat sequence that has been highly amplified in *Pre. intermedia* and are specifically distributed among the *Pre. intermedia*-related species. These findings expand our understanding of the genetic features of *Pre. intermedia* and the roles of CTns and MTns in the evolution of bacteria.

## 1. Introduction

*Prevotella intermedia* is a Gram-negative, black-pigmented anaerobic bacterium that is classified into the genus *Prevotella* belonging to the phylum *Bacteroidetes*. This bacterium is frequently found in subgingival plaque from patients with periodontal diseases and is considered one of the periodontal pathogens (a member of the so-called ‘orange complex’).^1^ *Prevotella* is phylogenetically close to other periodontal pathogens, namely *Porphyromonas gingivalis* and *Tannerella forsythia,* which are classified into the same phylum*. Prevotella intermedia* has also been associated with other oral infections, including endodontic infections,^2,3^ pregnancy gingivitis^4^ and acute necrotizing ulcerative gingivitis.^5^ An association of periodontitis with systemic diseases, such as cardiovascular diseases^6^ and preterm birth,^7^ has also been suggested, because the DNA of periodontal pathogens, including *Pre. intermedia*, has been detected in atherosclerotic plaques.^8^ In addition, the anti-*Pre. intermedia* IgM titre in umbilical cord blood is significantly higher in preterm infants than in full-term infants.^9^

The virulence factors of *Pre. intermedia*, such as adhesins,^10,11^ proteolytic enzymes^12^ and lipopolysaccharides,^13^ have been investigated, but its pathogenicity has not yet been fully elucidated. Three *Pre. intermedia* genome sequences, namely those from strains 17, ATCC25611 and ZT, are available, and an intra- and inter-species genomic comparison was previously performed to obtain information regarding the conservation and variation in the gene contents.^14^ However, the complete genome sequence, which may provide more precise information on the genome structure, gene content, mobile genetic elements (MGEs) ,and repetitive elements and therefore a deeper understanding of the evolutionary process of bacteria, has only been determined for strain 17.

In this study, we determined the complete sequences of two chromosomes in *Pre. intermedia* strain OMA14 and performed a fine comparison of the fully sequenced genomes of strains OMA14 and 17. Our analysis revealed an unusual amplification of conjugative and mobilizable transposons (CTns and MTns) and identified a novel repeat specific to a sublineage of the genus *Prevotella*, which was named *‘Pre. intermedia–nigrescens group-*specific repeat’. These MGEs and repeats were found to be deeply involved in the genomic rearrangements and the generation of the variability in the gene contents detected between the two genomes, particularly those associated with the second chromosome. These data will expand our understanding of the genetic features of *Pre. intermedia* and provide novel insights into the evolutionary process through which this bacterium has adapted to and survive in the stressful and variable oral habitat.

## 2. Materials and methods

### 2.1. Genome sequencing and sequence analysis

The strains used in this study are listed in Supplementary Table S1. *Prevotella intermedia* OMA14 was isolated from a periodontal pocket of a Japanese patient with periodontitis, and strain 17 was also isolated from a human periodontal pocket.^15^ The *Prevotella* strains were cultured anaerobically (10% CO_2_, 10% H_2_, and 80% N_2_) on trypto-soya agar (Nissui, Tokyo, Japan) supplemented with 0.5% brain–heart infusion, 0.1% cysteine, 5 μg/ml hemin, and 0.5 μg/ml menadione. Cells were harvested after 1 day of culture on trypto-soya agar plates to prepare genomic DNA as described previously.^16^

The *Pre. intermedia* OMA14 genome was sequenced using the Roche 454 GS FLX titanium platform (Roche Diagnostics, Indianapolis, IN, USA). In total, 363,377 single-end reads (150 Mb) were assembled into 85 contigs using the GS Assembler software version 2.6. For gap closing, a fosmid library was constructed using a Copy Control Fosmid Library Production kit (Epicentre, Madison, WI, USA) according to the manufacturer's instructions. Gaps were closed through the sequencing of gap-spanning PCR products and fosmid clones using an ABI3130xl DNA sequencer (Applied Biosystems, Foster City, CA, USA). To correct sequencing errors, the OMA14 genome was resequenced using the SOLiD (Applied Biosystems) and MiSeq (Illumina, San Diego, CA, USA) platforms. The candidate sequence errors were then confirmed by Sanger sequencing. The accuracy of sequence assembly was confirmed by optical mapping (Hitachi Solutions, Ltd, Tokyo, Japan).

Protein-coding sequences (CDSs) were identified and annotated using the Microbial Genome Annotation Pipeline (http://www.migap.org/)^17^ followed by manual curation using additional information obtained by a search of the Pfam protein family database (pfam.xfam.org).^18^ CDS numbers headed by ‘PIOMA14_I_’ or ‘PIOMA14_II_’ were assigned to each CDS on the large and small chromosomes, respectively. The Ori-Finder program^19^ was used to find *oriC* using the *Escherichia coli* DnaA boxes and one unmatch site option. The GenomeMatcher program^20^ was used to define the conserved genomic regions, inversions, and translocations between the two genomes using the bla2seq comparison at sensitivity 500 and 95% nucleotide identity threshold. The CD-HIT Suite for Biological Sequence Clustering and Comparison (http://weizhong-lab.ucsd.edu/cdhit_suite/cgi-bin/index.cgi)^21^ was used to identify orthologous CDS clusters (with cut-offs of >60% minimal alignment coverage and >90% sequence identity). The annotated genome sequences of strain OMA14 have been deposited into the DDBJ/NCBI/EMBL database under the Accession Numbers AP014597 and AP014598.

### 2.2. PCR assay for the excision of CTns and MTns

Primer pairs were designed to amplify the attachment site-containing regions of the excised and circularized forms of each CTn and MTn (we refer to attachment sites on CTns and MTns as ‘*attTn*’ in this manuscript) and the *attB* regions of each cast-off genome (Supplementary Table S2). PCR amplification was performed with 100 ng of genomic DNA and an LA Taq PCR kit (Takara Bio, Otsu, Japan) according to the manufacturer's instructions using the following temperature program: 94°C for 1 min and 30 cycles of 94°C for 20 s, 55°C for 30 s, and 68°C for 2 min. The amplified fragments were subjected to direct sequencing on an ABI PRISM 3130xl sequencer to determine the nucleotide sequences of the *attTn-* or *attB*-flanking regions.

### 2.3. *Prevotella intermedia/nigrescens* group-specific repeat-specific PCR

The *Pre. intermedia/nigrescens* group-specific repeat (PINSR)-specific primers PINSR-F and PINSR-R (Supplementary Table S2) were designed from the consensus sequence of PINSR. The expected product length was 149 bp. PCR amplification was performed with 50 ng of genomic DNA and an Ex Taq PCR kit (Takara Bio) using the following temperature program: 94°C for 1 min and 30 cycles of 94°C for 20 s, 51°C for 30 s, and 72°C for 30 s. The amplified products were detected by agarose gel electrophoresis followed by ethidium bromide straining. As a control, the 16S-rRNA gene was amplified using the primers 16S-rRNA-10F and 16S-rRNA-800 (Supplementary Table S2).

## 3. Results and discussion

### 3.1. General features of the *Pre. intermedia* genomes

**Figure 1.**
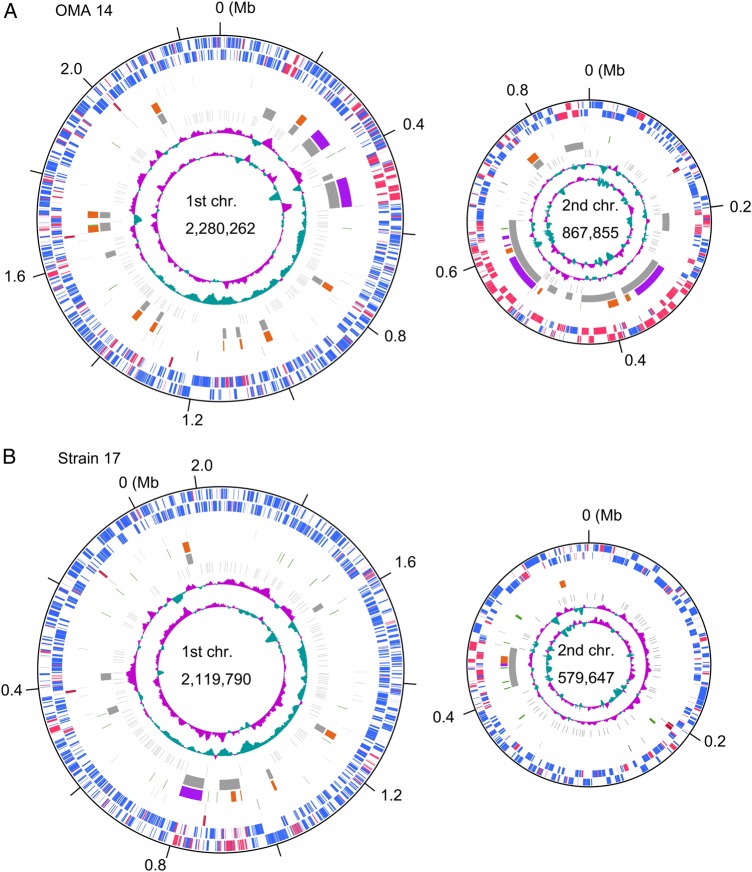
Circular maps of the chromosomes of *Prevotella intermedia* strains OMA14 and 17. The chromosomes of *Pre. intermedia* strains OMA14 and 17 are shown in A and B, respectively. The first and second circles (counted from the outside in) indicate CDSs on the plus and minus strands, respectively. The conserved and strain-specific CDSs are indicated in blue and red, respectively. The third to fifth circles show rRNA operons (red), tRNA genes (black), and MGEs (green, IS elements; purple, CTn; orange, MTn), respectively. In the sixth circle, large strain-specific segments are indicated in grey (>10 kb). The seventh circle shows PINSR (black). The GC skew [(G − C)/(G + C)] and GC content are shown in the eighth and ninth circles, respectively. Note that the *dnaA* genes of both strains are placed at the top of the first chromosome circle so that the three *rrn* operons of the two strains are located at analogous positions in this circular map presentation.

Similar to the genome of *Pre. intermedia* strain 17 and the other fully sequenced genomes of *Prevotella* species (*Prevotella denticola* and *Preotella melaninogenica*), the genome of OMA14 consisted of two circular chromosomes with sizes of 2,280,262 and 867,855 bp (Fig. 1 and Table 1). The large and small chromosomes of strain 17 were designated Chromosome II and Chromosome I (Accession No. NC_017861, NC_017860), respectively. However, to avoid confusion, we refer to the large and small chromosomes of both strains in this manuscript as the first and second chromosomes, respectively. The first nucleotide positions of the first and second chromosomes were assigned to the first nucleotides of the *dnaA* gene (PIOMA14_I_0001) and the initiation factor 3 gene (PIOMA14_II_0001), respectively. The first chromosome of OMA14 is larger than that of strain 17 (2,280 kb versus. 2,013 kb), and the second chromosome showed a more striking variation in size, namely 868 kb in OMA14 and 579 kb in strain 17 (Table 1 and Fig. 1). This variation is mainly due to the insertion of more MGEs into the second chromosome of OMA14 than into that of strain 17, as described in the next section. In both strains, although clear GC skew transitions were not detected in the second chromosome, clear transitions were observed at two loci located at opposite sites on the first chromosome. These two loci are located at analogous regions between the two strains. One of the loci (positions 1,498,452–1,498,927 in OMA14) contains multiple DnaA boxes and was predicted to be *oriC* of the first chromosome by Ori-Finder. Thus, this locus and the opposite locus most likely represent the replication origin and terminus of the first chromosome, respectively, but the *dnaA* gene is not located near the former locus. We have confirmed in the phylum *Bacteroidetes*, the *oriC* region is commonly located far from the *dnaA* gene (DoriC: an updated database of bacterial and archaeal replication origins, http://tubic.tju.edu.cn/doric/).^22^ PIOMA14_II_0001 on the second chromosome encodes a protein belonging to the Rep_3 superfamily. This may suggest a possibility that the second chromosome has a plasmid-type replication system. But, we did not find any genes related to plasmid maintenance and iteron-like repeat sequences in the vicinity of this CDS. Therefore, the replication origin and terminus of the second chromosome are currently unknown.

**Table 1.** General genomic features of *Prevotella intermedia* strains OMA14 and 17

Strain	OMA14			17		
Chromosome	First	Second	Total	First	Second	Total
Size (bp)	2,280,262	867,855	3,148,117	2,119,790	579,647	2,699,437
CDSs (strain-specific CDSs)	1,996 (24.7%)	801 (54.4%)	2,797 (33.2%)	1,796 (18.7%)	470 (27.2%)	2,266 (20.4%)
rRNA operons	3	1	4	3	1	4
tRNA genes	37	11	48	38	12	50
IS elements	8 (2)	6 (3)	14 (5)	17	10 (2)	27 (2)
CTns	2	4 (2)	6 (2)	1	1 (1)	2 (1)
MTns	10 (2)	5 (2)	15 (4)	5 (2)	2 (1)	7 (3)
PINSRs	120	57	177	122	62	184

The numbers in parentheses indicate the copy numbers of partial elements.

The OMA14 genome contains 2,797 CDSs, which is a greater number than that found in strain 17, reflecting the difference in genome size observed between the strains. In both strains, three and one rRNA operons are present in the first and second chromosomes, respectively. A full set of tRNA genes specifying all 20 amino acids was identified in both strains, i.e. 48 and 50 genes in strains OMA14 and 17, respectively. These tRNA genes are distributed to both chromosomes, but the tRNA^Ser^ genes (two copies) are encoded only on the second chromosome in both strains. A similar, small chromosome-specific distribution of specific tRNA genes has been observed in other *Prevotella* species with two chromosomes: tRNA^Cys^ and tRNA^Lys^ in *Pre. dentalis* (Accession No. NC_019960, NC_019968) and tRNA^Asp^, tRNA^Glu^, and tRNA^Ser^ in *Pre. melaninogenica* (Accession No. NC_014370, NC_014371). In addition to the rRNA operons and tRNA genes, the second chromosome of *Pre. ntermedia* contains many CDSs for house-keeping functions, including multiple ribosomal proteins (S1, L27, L28, and L33), multiple DNA polymerase III subunits (beta, gamma/tau, and epsilon), the RecA and RecN proteins, NADH dehydrogenase, NADH : ubiquinone oxidoreductase, prolyl-tRNA synthetase, heat-shock protein 90, and transcription termination factor Rho. Our preliminary analysis of other fully sequenced *Prevotella* species, *Pre. dentalis and Pre. melaninogenica*, indicate that many essential house-keeping genes are located in their second (smaller) chromosomes (ribosomal protein genes, chaperon protein genes, and many others). These data indicate that in *Prevotella* species, genes that are essentially required for cell growth are distributed in both the large and small chromosomes.

Clustered regularly interspaced short palindromic repeats (CRISPRs) were also found in both chromosomes but only the locus on the first chromosome contains CRISPR-associated genes. In OMA14, the numbers of repeats in the repeat/space regions of the CRISPR-47-29 locus on the first chromosome and CRISPR 36-30 locus on the second chromosome were 33 and 6, respectively, whereas the analogous regions in strain 17 contain 38 and 17 repeats, respectively. Notably the *cas1* gene of strain OMA14 has been disrupted by the insertion of a 34.2-kb phage-related segment, resulting in the apparent inactivation of the CRISPR system in OMA14. Smaller numbers of repeats in both loci of strain OMA14 (compared with strain 17) may be associated with the inactivation of the CRISPR system in this strain.

### 3.2. Mobile genetic elements

One of the notable genomic features of *Pre. intermedia* is the abundance of MGEs, particularly CTns and *MTns* (Table 1 and Supplementary Tables S3–S5). Thus, we conducted a detailed analysis of these MGEs in strains OMA14 and 17.

#### 3.2.1. IS elements

In total, 14 and 27 IS elements (including partial copies) were identified in strains OMA14 and 17, respectively. These elements were classified into two types, namely IS*Pi1* and IS*Pi2,* and both types are distributed to both chromosomes and are more abundant in strain 17 (Supplementary Tables S3 and S4). IS*Pi1* is closely related to IS*Pg3* found in *Por. gingivalis*, sharing the same inverted repeat sequences and generating the same length of target sequence duplication (7 bp) upon transposition. IS*Pi2* was assigned to the IS*4* family by IS finder (https://www-is.biotoul.fr/)^23^ using the tblastx program and default settings.

#### 3.2.2. CTn and MTn

**Figure 2.**
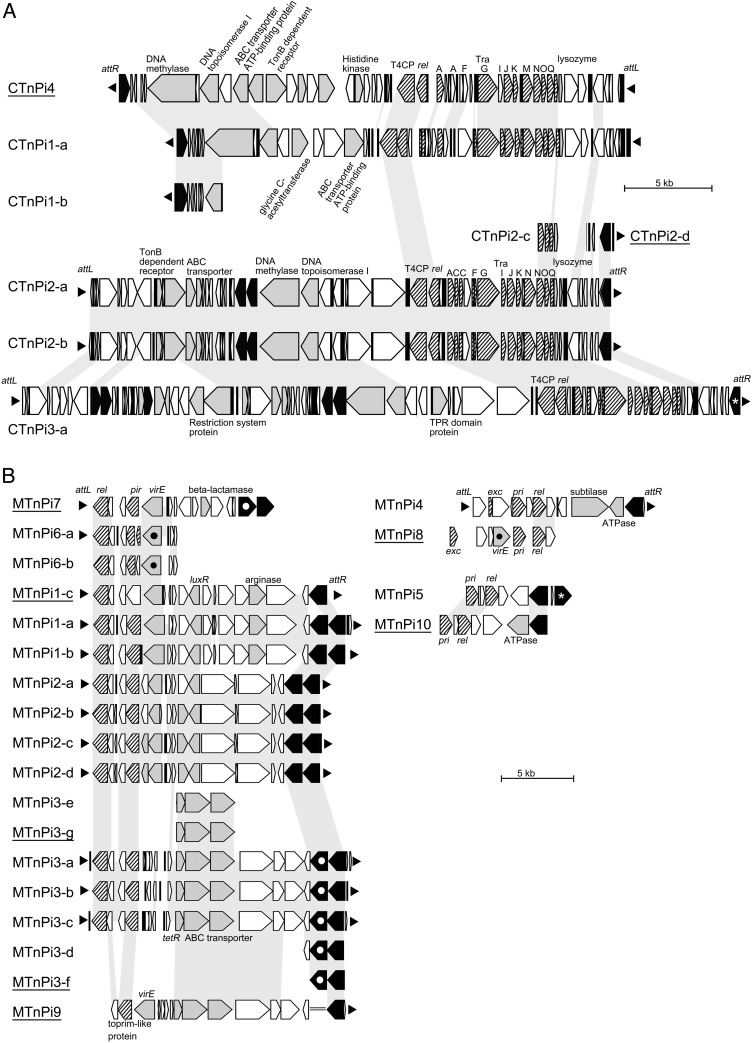
CTns and MTns identified in the genomes of strains OMA14 and 17. The gene organizations of all CTn and MTn copies identified in the two strains are shown (CTns in A and MTns in B). Some CTns or MTns (indicated by underlining) were found in strain 17, and others were found in strain OMA14. The CDSs are depicted by arrows (striped arrow, genes related to conjugation or mobilization; black arrow, integrase genes; grey arrow, other functionally annotated CDSs; and white arrow, genes for hypothetical proteins). The black triangles indicate the *att* sites of each CTn and MTn. Three *att* sequences (*attL* of MTnPi6-a, *attL* of MTnPi7, and *attR* of MTnPi9) were predicted based on the sequence homology to the consensus *att* core sequence of MTnPi1, MTnPi2, and MTnPi3 (see Supplementary Table S3). CTn and MTn transposons lacking either one of *att* sequences can be regarded as the remnants. The grey shading indicates homologous CDSs with >70% amino acid sequence identity. The CDS groups indicated by asterisks, open circles, and black circles exhibited >50% identity among the group. T4CP, coupling protein T4PC; rel, relaxase; pri, primase family protein; exc, excisionase; tetR, TetR family transcriptional regulator; Lux, LuxR family transcriptional regulator. A double line indicates a degraded integrase gene.

OMA14 contains 6 CTns and 15 MTns, whereas strain 17 possesses 2 CTns and 7 MTns (including highly degraded MTns; Fig. 2 and Supplementary Tables S3 and S5). These transposons account for 14.6 and 4.8% of the total genomes of OMA14 and strain 17, respectively. In addition, CTns and MTns occupy large portions (20.5%) of the second chromosome, particularly in OMA14, which is one of the main driving forces responsible for the marked difference in the size of the second chromosomes between the two strains. The CTns and MTns identified were classified into four (CTnPi1–CTnPi4) and 10 types (MTnPi1–MTnPi10), respectively. Among these, one type of CTn and two types of MTns are shared by the two strains, suggesting that these CTn and MTns may be widely distributed among *Pre. intermedia* strains. Several copies found in OMA14 (CTnPi2-a and -b; MTnPi1-a and -b; MTnPi2-a, -b, -c, and -d; and MTnPi3-a, -b, and -c) are nearly identical and thus appear to have been very recently duplicated (Fig. 2).

Both CTnPi1 (found in OMA14) and CTnPi4 (strain 17) belong to the CTnPg1 family, which was originally identified in *Por. gingivalis*,^16^ and share a set of backbone genes for integration- and conjugal transfer-related functions with other family members, which have been identified in *Prevotella*, *Porphyromonas*, and *Bacteroides* species, all belonging to the order *Bacteroidales* (Supplementary Fig. S1). Both CTns, particularly CTnPi4, carry various accessory genes. Some of these genes are distributed in other CTnPg1 family members, e.g. genes for an ABC transporter component and a glycine C-acetyltransferase (CTnPi1) in the family members found in *Por. gingivalis* TDC60 and *Pre. endodontalis* ATCC 35406 and genes encoding an ABC transporter component, a TonB-dependent receptor, and several other proteins (CTnPi4) in the members found in *Pre. nigrescens* ATCC 33563 and *Prevotella buccae* D17. These results suggest that accessory genes are frequently exchanged between CTnPg1 family members.

CTnPi2 and CTnPi3, both of which are found in OMA14, are closely related to one another, sharing a conserved backbone encoding integration- and conjugal transfer-related functions, which are partially homologous to those of CTnPi1 and CTnPi4 (Fig. 2A). CTnPi2 and CTnPi3 contain multiple integrase genes in internal regions. There are no putative *att* sequences just upstream of those internally residing integrase genes, but these integrases contain the motifs conserved in functional tyrosine recombinases. It is possible that the two CTns were generated by the insertion of at least one integrative element similar to MTns, i.e. one element in CTnPi2 and two or three elements in CTnPi3 with one shared by the two CTns. As accessory genes, CTnPi3 carries genes for a type I restriction-modification system and a tetratricopeptide repeat domain-containing protein as well as genes that are shared with CTnPi2 (encoding an ABC transporter component and a TonB-dependent receptor).

MTns are a group of transposons that contain an *oriT* sequence and can thus be mobilized by a co-resident conjugative plasmid or transposon.^24^ Among the 10 types of MTns identified in the two strains, only degraded copies were found for several types (MTnPi5, 6, 7, 8, 9, and 10). However, genes for relaxase, a primase family protein, and integrase, which are required for MTn function, were found in most types (Fig. 2B). Because the integrases of all MTns are tyrosine recombinases, they all belong to type I MTn.^24^ MTnPi1, 2, 3, 6, 7, and 9 appear to be related to one another and share similar relaxases, primase family proteins, and integrases. MTnPi4 and 8 and MTnPi5 and 10 appear to form different groups, respectively. A unique feature of this group is the presence of two integrase genes located in tandem, although the second gene (a downstream copy) is missing or degraded in some MTn copies. The two integrase genes in each MTn show no significant homology to one another (see the next subsection for a discussion of the functions of these two integrases). It was previously reported that Tn1806 of *Streptococcus pneumonia* AP200 also contains two *int* genes in tandem.^25^ The other four MTns may be classified into two groups: one including ‘MTnPi4 (found in OMA14) and MTnPi8 (strain 17)’, and the other including ‘MTnPi5 (OMA14) and MTnPi-10 (strain 17)’, because each pair shares similar genes for a primase family protein, relaxase, or integrase. Most MTns also carry one or more accessory genes, such as those encoding an ABC transporter component, a LuxR family transcriptional regulator, a subtilase-like protease, a β-lactamase family protein, and an arginase.

#### 3.2.3. Excision assay of CTns and MTns and identification of attachment sequences

**Figure 3.**
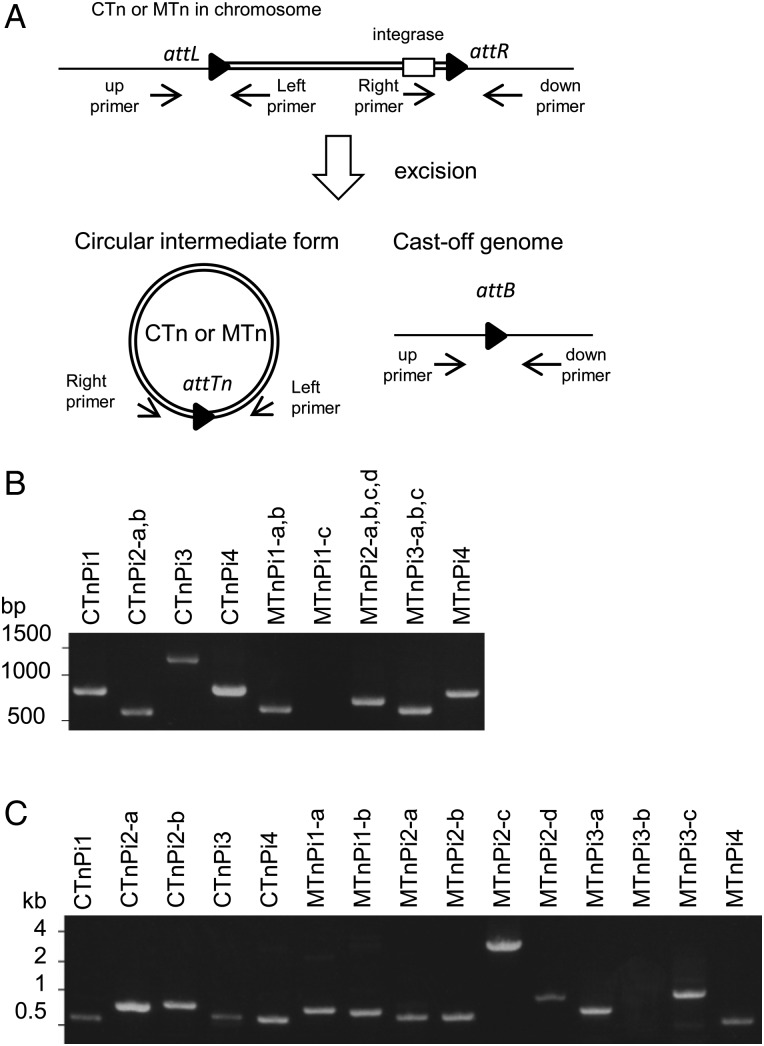
Excision of CTns and MTns. (A) Schematic representation of the excision of CTns and MTns from the chromosome. Black triangles indicate the *att* sites. The PCR primers are indicated by arrows. Agarose gel electrophoresis of the PCR products obtained by the primer pairs left/right (B) and up/down (C). The *attTn*-containing regions in CTnPi-4 and MTnPi1-c were amplified from strain 17. The other regions were amplified from OMA14. *attTn* indicates the *att* sequence on the excised and circularized form of transposon.

In general, CTns/MTns are integrated into a specific target site (attachment site; *att*) that contains a short sequence specific to each CTn or MTn. Upon integration, the *att* sequences are duplicated and form chromosome/CTn (or MTn) boundaries (*attL* and *attR*), which are good landmarks for the definition of each CTn/MTn. However, it is not always easy to identify *att* sequences. Therefore, we used PCR assays to detect the excised and circularized intermediates of each CTn and MTn and thereby examined whether the identified CTns and MTns are excised from the chromosome (Fig. 3A). In addition, through a sequencing analysis of the PCR products, we determined the *att* sequences of each CTn/MTn (*attTn* on the excised and circularized forms of transposon and *attL* and *attR* at chromosome/transposon boundaries). Through this series of analyses, we found that CTnPi1, 2, 3, and 4 and MTnPi1, 2, 3, and 4 are constitutively excised from the chromosome to form a circular intermediate (Fig. 3B), and we identified their *att* sequences (Supplementary Fig. S2 and Table S3). Among these CTns and MTns, multiple copies of CTnPi2 and MTnPi1, 2, and 3 were found. Because the sequences of *attTn*-flanking regions in the different copies are identical, it was impossible to determine which copy is excised in each type of transposon. We therefore performed an additional PCR assay to detect the chromosomal attachment sites (*attB*) of each copy from which CTn/MTn has been excised (Fig. 3A and C). This analysis confirmed that all of the copies, with the exception of one copy of MTnPi1 and one copy of MTnPi3, were excised from the chromosome. These results suggest that these CTns and MTns are likely active. We speculate that they can potentially transfer (or be transferred) to other bacteria, but conjugation assays for each transposon need to confirm their activities. We were unable to detect the excision of the remaining MTns (data not shown), which was expected for six of the MTns (MTnPi5–MTnPi10) due to their high degree of degradation.

Notably, MTnPi1-c was not excised (Fig. 3B), which was rather unexpected, because this element contains all of the genes found in other MTnPi1 copies except for the second (downstream) integrase (Fig. 2). The integrase of MTnPi1-c has been significantly diverged in sequence from the upstream integrase of MTnPi1-a and MTnPi1-b (92.7% amino acid sequence identity). This makes a sharp contrast to the fact that the upstream integrases of MTnPi1-a and MTnPi1-b are identical in amino acid sequence. Although the motif for tyrosine recombinase is conserved in the integrase of MTnPi1-c, amino acid substitutions have accumulated in the C-terminal region. Inactivation of the integrase by these amino acid substitutions may be a possible reason for the failure of excision of MTnPi1-c. However, the apparent and specific deletion of the downstream integrase gene from MTnPi1-c suggests another possibility that both integrases may be required for their function, at least for the excision of MTnPi1. If this is true, two integrase genes in MTnPi2 and MTnPi3 would also be required for the excision of these MTns, because their integrases show high sequence similarities to those of MTnPi1 (Fig. 2).

In addition to allowing the exact definition of chromosome/CTn (or MTn) boundaries, the analysis of the identified *att* sequences/sites yielded several important findings. CTnPi1 and CTnPi4 use the same 13-bp sequence (TTTTCNNNNAAAA) that used by other CTnPg1 family members as its attachment site, supporting our conclusion regarding the relationship of these two CTns to the CTnPg1 family obtained from the previous structural comparison (Fig. 2). Second, CTnPi2-b, CTnPi3, and MTnPi4 are inserted into tRNA genes, similar to several other CTns^24^ and MTns.^26,27^ Another copy of CTnPi2-a is inserted into an integrase gene (II_0226) that may form part of a degraded MTn similar to MTnPi1 and MTnPi2. Notably, MTnPi1, 2, and 3 use similar 11-bp sequences as the *att* site, supporting the close relatedness of these MTns that was inferred from the previous structural comparison (Fig. 2).

### 3.4. *Prevotella intermedia/nigrescens* group-specific repeat

**Figure 4.**
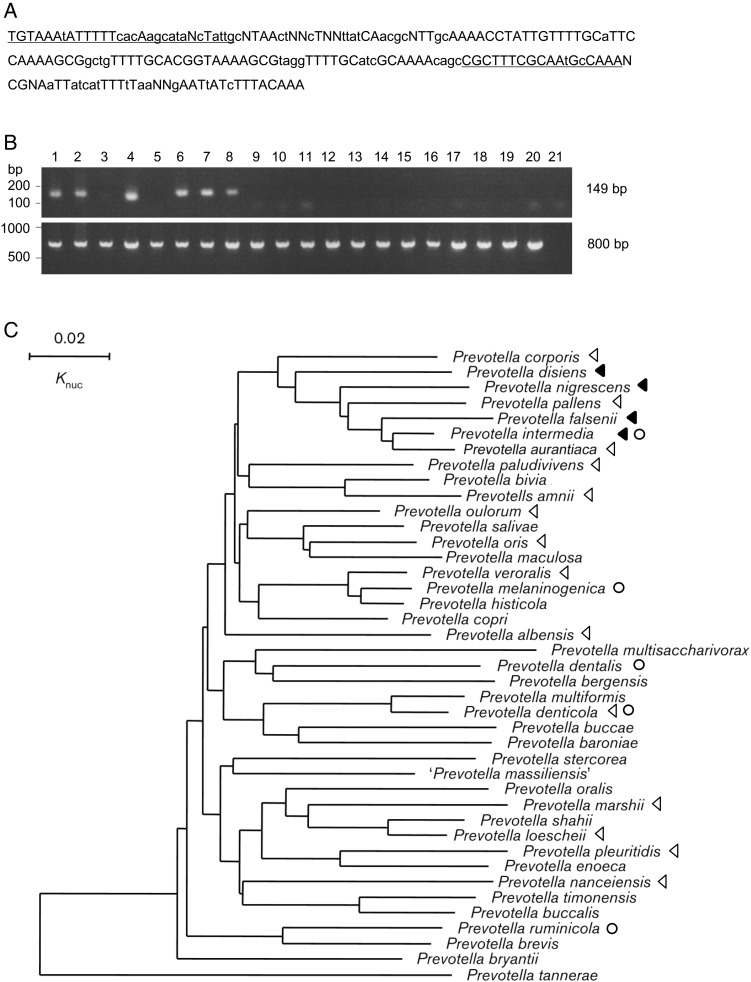
*Prevotella intermedia–nigrescens* group-specific repeat (PINSR). (A) The 188-bp consensus sequence denoted PINSR was deduced from the alignment of all PINSR copies found in strains OMA14 and 17. The nucleotides conserved in >90% and >50% copies are indicated in upper case and lower case, respectively. The target sequences of the designed PCR primers are underlined. (B) PCR detection of PINSR sequences (upper) and 16S-rRNA sequences (lower) in various *Prevotella* species. Lane 1*, Pre. intermedia* OMA14; Lane 2, *Pre. intermedia* strain 17; Lane 3, *Pre. aurantiaca* OMA31; Lane 4, *Pre. falsenii* JCM15124T; Lane 5, *Pre. pallens* AHN10371T; Lane 6, *Pre. nigrescens* ATCC33563; Lane 7, *Pre. nigrescens* ATCC25261; Lane 8, *Pre. disiens* ATCC29426; Lane 9, *Pre. corporis* ATCC33547; Lane 10, *Pre. paludivivens* JCM 13650T; Lane 11, *Pre. amnii* CCUG53648; Lane 12, *Pre. oulorum* ATCC43324; Lane 13, *Pre. oris* ATCC33573; Lane 14, *Pre. veroralis* ATCC33779; Lane 15, *Pre. albensis* DSM 11370; Lane 16, *Pre. denticola* ATCC35308; Lane 17, *Pre. marshii* DSM16973; Lane 18, *Pre. loesheii* ATCC15930; Lane 19, *Pre. pleuritidis* JCM 14110 T; Lane 20, *Pre. nanceiensis* JCM 15639T; and Lane 21, distilled water (negative control). (C) Distribution of PINSR within the genus *Prevotella*. The presence (closed triangles) or absence (open triangles) of PINSR is shown in the 16S-rRNA gene sequence-based phylogenetic tree of the genus *Prevotella* (adapted from Sakamoto et al.^30^). Species, the complete genome sequences of which are available in public databases, are indicated by open circles. Only the species indicated by triangles were examined for the presence of PINSR by PCR.

We found a 188-bp incomplete repeat sequence located in intergenic regions in the genomes of both strains OMA14 and 17 (Fig. 4A). This repeat sequence was expanded to 177 copies in OMA14 and 184 copies in strain 17 (Supplementary Table S6). A database search for homologous sequences confirmed that this 188-bp repeat is also present in the downstream region of the *tnaA* gene^28^ and DNA probe Pig27^29^ of *Pre. intermedia* ATCC 25611 and the draft genomes of *Pre. nigrescens* ATCC 33563 and F103. We further examined 18 *Prevotella* species (Supplementary Table S1) for the presence of this repeat by PCR using primers specific to the repeat, which were designed based on the consensus sequence deduced from all copies found in strains OMA14 and 17 (Fig. 4A). This analysis revealed that, in addition to *Pre. intermedia* and *Pre. nigrescens*, *Prevotella falsenii* and *Prevotella disiens* also contain the repeat (Fig. 4B). These four species are closely related and form a clade in the genus *Prevotella* (Fig. 4C).^30,31^ Although some species in this clade lack the repeat, none of the *Prevotella* species belonging to other clades apparently possess the 188-bp repeat. We therefore named this repeat PINSR (*Prevotella intermedia/nigrescens* group-specific repeat). The selective distribution of PINSR in the *Pre. intermedia/nigrescens* group suggests that PINSR was acquired by a common ancestor of this group and then amplified in some species (particularly *Pre. intermedia*) or deleted (or diverged in sequence) from other species following speciation. PINSR-specific PCR would thus be useful for identifying *Pre. intermedia* and the three other PINSR-possessing *Prevotella* species.

In addition, small CDSs that overlap PINSR were annotated in the genome sequence of strain 17 (Accession No. NC_017860, NC_017861). However, clear ribosome-binding sequences are not present in most cases, and no significant homology to known proteins was detected. The sizes of these small CDSs are highly variable. Therefore, it is most likely that PINSR does not contain protein-coding sequences. PINSR also does not contain terminal-inverted repeat sequences. Therefore, PINSR is distinct from the miniature-inverted repeat transposable element (MITE) that is transposable by the action of the transposase provided *in trans* from a cognate IS element. Intriguingly, approximately three-quarters of the PINSR-containing regions identified in strains OMA14 and 17 are conserved between the two strains, suggesting that PINSR may have been amplified prior to the separation of the two strains. This hypothesis is supported by the finding that most strain-specific regions, such as CTns and MTns, in both strains do not contain PINSR. Although PINSR apparently played some roles in the genome rearrangement that occurred between the two strains, as described in the next section, further studies are necessary to elucidate the biological functions and source of PINSR (if present).

### 3.5. Genome rearrangement

A whole-genome sequence alignment analysis revealed that extensive genomic rearrangements have occurred between strains OMA14 and 17. We identified 26 inversions/translocations and >100 simple insertions/deletions (Supplementary Fig. S3). Approximately two-thirds of the identified inversions/translocations are associated with MGEs, and at least one IS, CTn, or MTn element is present at each inversion/translocation site. The remaining inversions/translocations occurred between PINSRs, *rrn* operons, or tRNA genes. This analysis also identified strain-specific segments (>10 kb) in both genomes, namely Segments A-W in OMA14 and Segments A-I in strain 17 (the sixth circle in Fig. 1, Supplementary Tables S7 and S8). Approximately half of these segments simply consist of a single CTn or MTn (10 of the 23 segments in stain OMA14 and 4 of the 9 segments in strain 17). However, several segments, particularly larger ones, exhibit composite natures containing multiple MGTs. Furthermore, several segments contain phage-related genes and thus likely represent prophages or degraded prophages. These features further support the hypothesis that MGEs played key roles in generating the genomic diversity of *Pre. intermedia*.

### 3.6. Gene repertoire comparison between the two *Pre. intermedia* strains

To compare the gene repertoires of strains OMA14 and 17, we performed a clustering analysis of all CDSs identified in the two strains using the CD-HIT program, which generated 2,884 CDS clusters. Among these, 1,744 were found to be conserved in both strains, whereas 686 and 454 are specific to strains OMA14 and 17, respectively (Supplementary Fig. S4). This number of conserved genes is somewhat larger than that recently revealed by the comparison of the genome of strain 17 and draft sequences of two *Pre. intermedia* strains (∼1,600).^14^ This difference may be due to the difference in the number of strains analysed and to the difference in the quality of the sequences or annotations (complete versus. draft sequences). Therefore, the core genome of *Pre. intermedia* most likely consists of 1,600–1,700 genes and encodes most virulence genes that have been identified in *Pre. intermedia*, such as proteolytic enzyme interpain A (II_0221)^12^ and the adhesins AdpC (I_1236)^10^ and AdpF (I_1057).^11^ The gene set encoding the type IX secretion system (T9SS), which was originally identified in *Por. gingivalis*,^32,33^ was also found in the core genome.

The strain-specific CDSs listed in Supplementary Tables S9 and S10 include many genes in CTns and MTns, reflecting the difference in the transposon repertoires between the two strains. In particular, as many as 54.4% of the CDSs on the second chromosome of strain OMA14, where CTns and MTns are most densely integrated, are strain-specific CDSs. Notably, each strain contains a large number of strain-specific genes related to DNA restriction modification. Another noteworthy finding is the presence of strain-specific glycosyltransferase gene clusters in each strain (Segment D in OMA14 and Segment G in strain 17), which suggests that the two strains may produce different extracellular polysaccharides.

### 3.7. Conclusions

In this study, we determined the complete genome sequence of *Pre. intermedia* OMA14 and compared it with the completely sequenced *Pre. intermedia* strain 17. Our analysis revealed that the genomes of *Pre. intermedia* are unusually enriched in CTns and MTns, which account for large parts of the strain-specific sequences observed in each *Pre. intermedia* genome. Together with IS elements, these CTns and MTns are also involved in the extensive genomic rearrangements observed between the two strains. In addition, we found a novel repeat element (named PINSR) that has been extensively amplified in *Pre. intermedia* and several *Prevotella* species belonging to the *Pre. intermedia*–*nigrescens* group. These results expand our understanding of the genetic features and genomic diversity of *Pre. intermedia* and provide novel insights into the roles of MGEs, particularly CTns and MTns, in the evolution of bacteria.

## Supplementary data

Supplementary data are available at www.dnaresearch.oxfordjournals.org

## Funding

This work was supported by a Grant-in-Aid for Scientific Research on Innovative Areas ‘Genome Science’ from the Ministry of Education, Culture, Sports, Science and Technology (No. 221S0002), and by grants from the Japan Society for the Promotion of Science (No. 20249073 and No. 24659817) to K.N. Funding to pay the Open Access publication charges for this article was provided by grants from the Japan Society for the Promotion of Science (No. 26670804) to K.N.

## Supplementary Material

Supplementary Data
