# Supplementary material for: The complete genome sequencing of Prevotella intermedia strain OMA14 and a subsequent fine-scale, intra-species genomic comparison reveal an unusual amplification of conjugative and mobile transposons and identify a novel Prevotella-lineage-specific repeat
Source: DNA Res. 2015 Dec 8;23(1):11–9. doi: 10.1093/dnares/dsv032 (PMC4755523; doi:10.1093/dnares/dsv032)
Supplement: Supplementary Data [file supp_23_1_11__index.html]

The complete genome sequencing of Prevotella intermedia strain OMA14 and a subsequent fine-scale, intra-species genomic comparison reveal an unusual amplification of conjugative and mobile transposons and identify a novel Prevotella-lineage-specific repeat — Supplementary Data 

# The complete genome sequencing of *Prevotella intermedia* strain OMA14 and a subsequent fine-scale, intra-species genomic comparison reveal an unusual amplification of conjugative and mobile transposons and identify a novel *Prevotella-*lineage-specific repeat

## Supplementary Data

Supplementary Data

- Supplementary Data - Doc file
- Supplementary Figure 1 - ppt file
- Supplementary Figure 2 - ppt file
- Supplementary Figure 3 - ppt file
- Supplementary Figure 4 - ppt file
- Supplementary Table 1 - xls file
- Supplementary Table 2 - xls file
- Supplementary Table 3 - xls file
- Supplementary Table 4 - xls file
- Supplementary Table 5 - xls file
- Supplementary Table 6 - xls file
- Supplementary Table 7 - xls file
- Supplementary Table 8 - xls file
- Supplementary Table 9 - xls file
- Supplementary Table 10 - xls file
