# Supplementary material for: The complete genome sequencing of Prevotella intermedia strain OMA14 and a subsequent fine-scale, intra-species genomic comparison reveal an unusual amplification of conjugative and mobile transposons and identify a novel Prevotella-lineage-specific repeat
Source: DNA Res. 2015 Dec 8;23(1):11–9. doi: 10.1093/dnares/dsv032 (PMC4755523; doi:10.1093/dnares/dsv032)
Supplement: Supplementary Data [file supp_dsv032_dsv032supp.doc]

Legends for the supplementary figures

Supplementary Figure S1. Comparison of CTnPi1-a, CTnPi4, CTnPg1 and CTnPg1-like CTns.

The location and directions of the CDSs (arrows) are drawn to scale. Homologous CDSs with >70% amino acid sequence identity are indicated with grey shading. The black triangles indicate *att* sites. The homologous accessory genes are indicated by single, double and dotted lines.

Supplementary Figure S2. Sequence alignment of the *att* sites.

The *attTn*, *attL* and *attR* regions of CTns (A) and MTns (B) are shown, and the common sequences in the *att* sites are underlined. “*attTn”* indicates the attachment site on the excised and circularized form of CTn and MTn.

Supplementary Figure S3. Plots of the DNA sequence identity of the first and second chromosomes of strains OMA14 and 17.

The *dnaA* gene in the first chromosome (A) and the *repB* gene in the second chromosome (B) are located at the bottom left corner. The closed circles indicate the mobile genetic elements (CTn, MTn, or IS) that mediated inversions and translocations. Other genetic elements that mediated inversions or translocations are indicated by open squares, (*rrn* operon), open triangles (CRISPR-associated gene), black triangles (PINSR), and open circles (tRNA gene). The locations of large strain-specific segments (>10 kb) found in strain OMA14 (Segments A-W) and strain 17 (Segments A-I) are also indicated.

Supplementary Figure S4. Venn diagram of the CDS comparison between strains OMA14 and 17.

All CDSs in each strain were clustered with cut-offs of >60% minimal alignment coverage and >90% sequence identity.
