# Supplementary material for: The complete genome sequencing of Prevotella intermedia strain OMA14 and a subsequent fine-scale, intra-species genomic comparison reveal an unusual amplification of conjugative and mobile transposons and identify a novel Prevotella-lineage-specific repeat
Source: DNA Res. 2015 Dec 8;23(1):11–9. doi: 10.1093/dnares/dsv032 (PMC4755523; doi:10.1093/dnares/dsv032)
Supplement: Supplementary Data [file supp_dsv032_dsv032supp_fig1.ppt]

## Slide 1
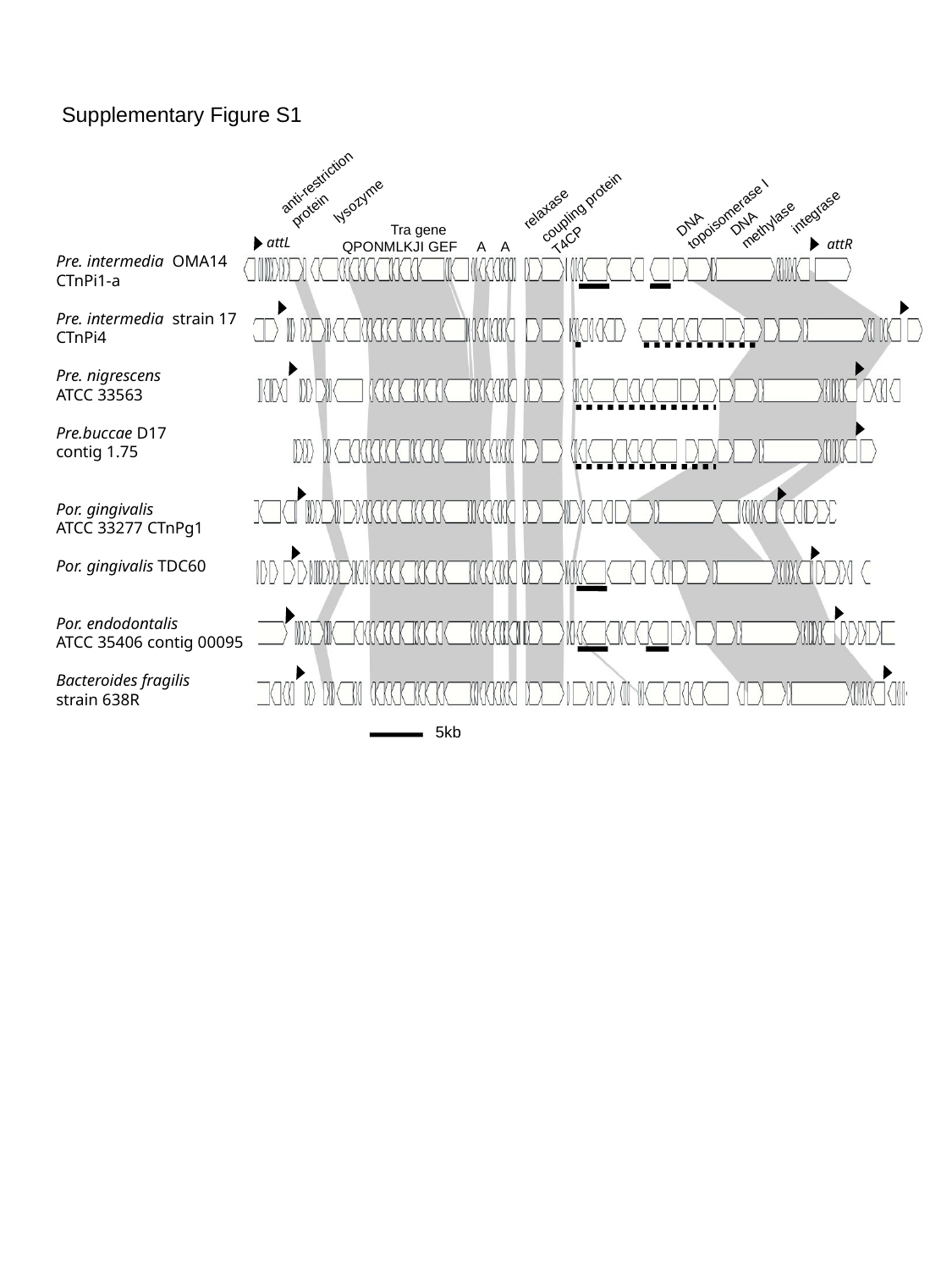

Supplementary Figure S1
anti-restriction
protein
DNA topoisomerase I
lysozyme
relaxase
coupling protein T4CP
DNA
methylase
integrase
 Tra gene
QPONMLKJI GEF A A
attL
attR
Pre. intermedia OMA14
CTnPi1-a
Pre. intermedia strain 17
CTnPi4
Pre. nigrescens
ATCC 33563
Pre.buccae D17
contig 1.75
Por. gingivalis
ATCC 33277 CTnPg1
Por. gingivalis TDC60
Por. endodontalis
ATCC 35406 contig 00095
Bacteroides fragilis
strain 638R
5kb
