# Supplementary material for: The complete genome sequencing of Prevotella intermedia strain OMA14 and a subsequent fine-scale, intra-species genomic comparison reveal an unusual amplification of conjugative and mobile transposons and identify a novel Prevotella-lineage-specific repeat
Source: DNA Res. 2015 Dec 8;23(1):11–9. doi: 10.1093/dnares/dsv032 (PMC4755523; doi:10.1093/dnares/dsv032)
Supplement: Supplementary Data [file supp_dsv032_dsv032supp_fig2.ppt]

## Slide 1
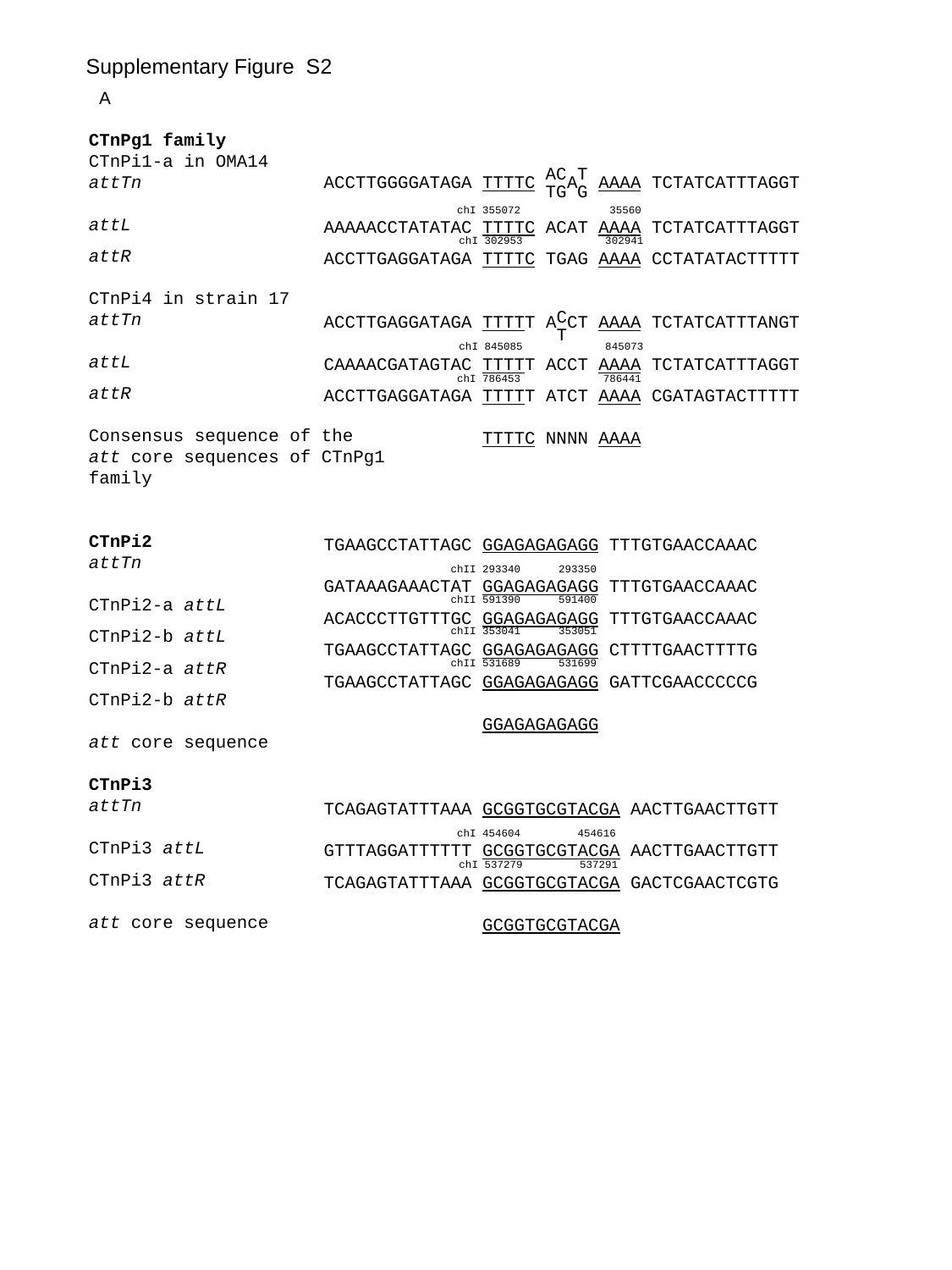

Supplementary Figure S2
A
CTnPg1 family
CTnPi1-a in OMA14
attTn
attL
attR
CTnPi4 in strain 17
attTn
attL
attR
Consensus sequence of the att core sequences of CTnPg1 family
CTnPi2
attTn
CTnPi2-a attL
CTnPi2-b attL
CTnPi2-a attR
CTnPi2-b attR
att core sequence
CTnPi3
attTn
CTnPi3 attL
CTnPi3 attR
att core sequence
AC T
TG G
ACCTTGGGGATAGA TTTTC A AAAA TCTATCATTTAGGT
 chI 355072 35560
AAAAACCTATATAC TTTTC ACAT AAAA TCTATCATTTAGGT
 	 chI 302953 302941
ACCTTGAGGATAGA TTTTC TGAG AAAA CCTATATACTTTTT
ACCTTGAGGATAGA TTTTT A CT AAAA TCTATCATTTANGT
 chI 845085 845073
CAAAACGATAGTAC TTTTT ACCT AAAA TCTATCATTTAGGT
 chI 786453 786441
ACCTTGAGGATAGA TTTTT ATCT AAAA CGATAGTACTTTTT
 TTTTC NNNN AAAA
TGAAGCCTATTAGC GGAGAGAGAGG TTTGTGAACCAAAC
 chII 293340 293350
GATAAAGAAACTAT GGAGAGAGAGG TTTGTGAACCAAAC
 chII 591390 591400
ACACCCTTGTTTGC GGAGAGAGAGG TTTGTGAACCAAAC
 chII 353041 353051
TGAAGCCTATTAGC GGAGAGAGAGG CTTTTGAACTTTTG
 chII 531689 531699
TGAAGCCTATTAGC GGAGAGAGAGG GATTCGAACCCCCG
 GGAGAGAGAGG
TCAGAGTATTTAAA GCGGTGCGTACGA AACTTGAACTTGTT
 chI 454604 454616
GTTTAGGATTTTTT GCGGTGCGTACGA AACTTGAACTTGTT
 chI 537279 537291
TCAGAGTATTTAAA GCGGTGCGTACGA GACTCGAACTCGTG
 GCGGTGCGTACGA
C
T

## Slide 2
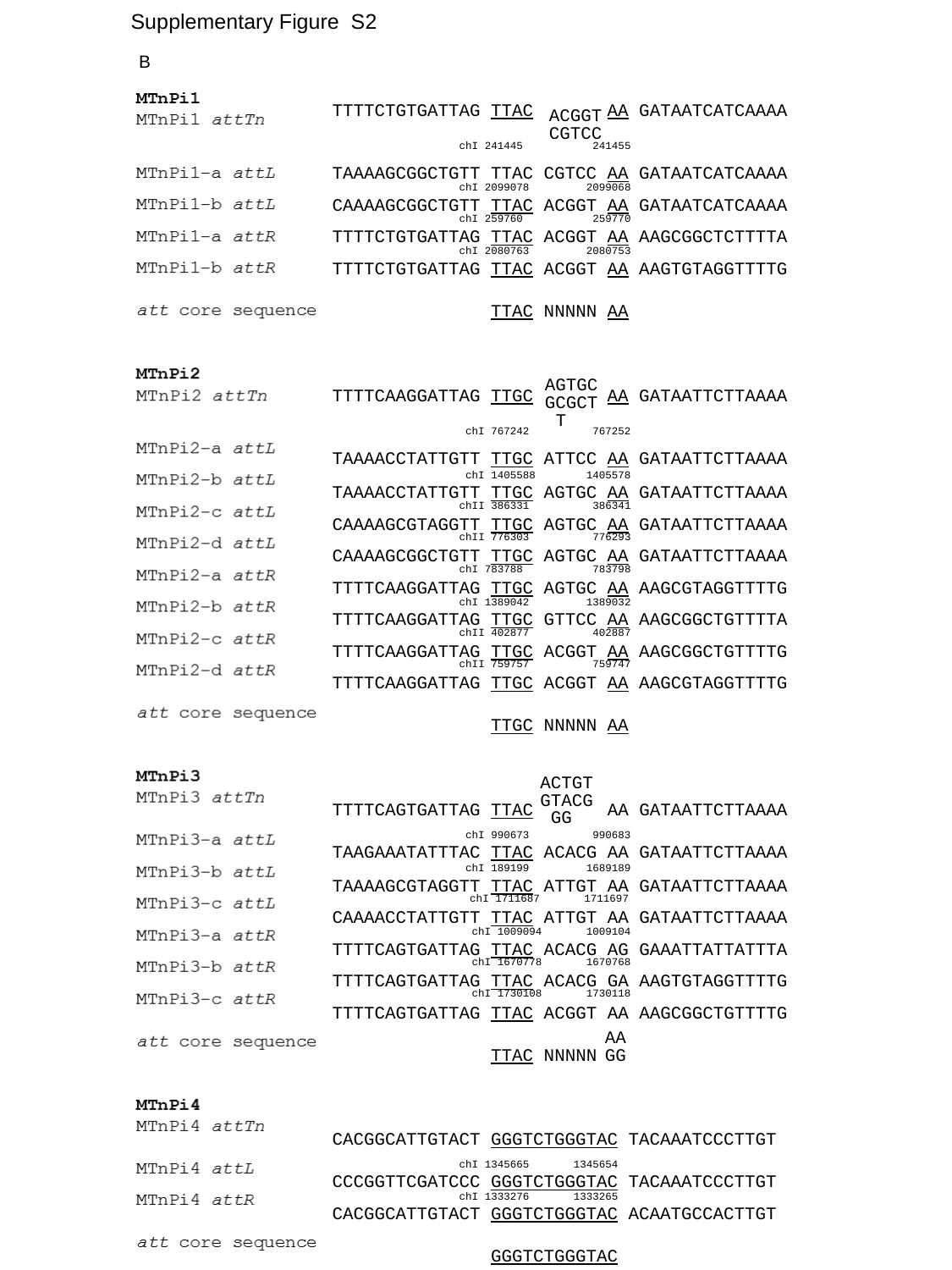

Supplementary Figure S2
B
TTTTCTGTGATTAG TTAC AA GATAATCATCAAAA
 chI 241445 241455
TAAAAGCGGCTGTT TTAC CGTCC AA GATAATCATCAAAA
 chI 2099078 2099068
CAAAAGCGGCTGTT TTAC ACGGT AA GATAATCATCAAAA
 chI 259760 259770
TTTTCTGTGATTAG TTAC ACGGT AA AAGCGGCTCTTTTA
 chI 2080763 2080753
TTTTCTGTGATTAG TTAC ACGGT AA AAGTGTAGGTTTTG
 TTAC NNNNN AA
TTTTCAAGGATTAG TTGC AA GATAATTCTTAAAA
 chI 767242 767252
TAAAACCTATTGTT TTGC ATTCC AA GATAATTCTTAAAA
 chI 1405588 1405578
TAAAACCTATTGTT TTGC AGTGC AA GATAATTCTTAAAA
 chII 386331 386341
CAAAAGCGTAGGTT TTGC AGTGC AA GATAATTCTTAAAA
 chII 776303 776293
CAAAAGCGGCTGTT TTGC AGTGC AA GATAATTCTTAAAA
 chI 783788 783798
TTTTCAAGGATTAG TTGC AGTGC AA AAGCGTAGGTTTTG
 chI 1389042 1389032
TTTTCAAGGATTAG TTGC GTTCC AA AAGCGGCTGTTTTA
 chII 402877 402887
TTTTCAAGGATTAG TTGC ACGGT AA AAGCGGCTGTTTTG
 chII 759757 759747
TTTTCAAGGATTAG TTGC ACGGT AA AAGCGTAGGTTTTG
 TTGC NNNNN AA
TTTTCAGTGATTAG TTAC AA GATAATTCTTAAAA
 chI 990673 990683
TAAGAAATATTTAC TTAC ACACG AA GATAATTCTTAAAA
 chI 189199 1689189
TAAAAGCGTAGGTT TTAC ATTGT AA GATAATTCTTAAAA
 chI 1711687 1711697
CAAAACCTATTGTT TTAC ATTGT AA GATAATTCTTAAAA
 chI 1009094 1009104
TTTTCAGTGATTAG TTAC ACACG AG GAAATTATTATTTA
 chI 1670778 1670768
TTTTCAGTGATTAG TTAC ACACG GA AAGTGTAGGTTTTG
 chI 1730108 1730118
TTTTCAGTGATTAG TTAC ACGGT AA AAGCGGCTGTTTTG
 TTAC NNNNN
CACGGCATTGTACT GGGTCTGGGTAC TACAAATCCCTTGT
 chI 1345665 1345654
CCCGGTTCGATCCC GGGTCTGGGTAC TACAAATCCCTTGT
 chI 1333276 1333265
CACGGCATTGTACT GGGTCTGGGTAC ACAATGCCACTTGT
 GGGTCTGGGTAC
ACGGT
CGTCC
AGTGC
GCGCT
 T
ACTGT
GTACG
 GG
AA
GG
