# Supplementary material for: The complete genome sequencing of Prevotella intermedia strain OMA14 and a subsequent fine-scale, intra-species genomic comparison reveal an unusual amplification of conjugative and mobile transposons and identify a novel Prevotella-lineage-specific repeat
Source: DNA Res. 2015 Dec 8;23(1):11–9. doi: 10.1093/dnares/dsv032 (PMC4755523; doi:10.1093/dnares/dsv032)
Supplement: Supplementary Data [file supp_dsv032_dsv032supp_fig3.ppt]

## Slide 1
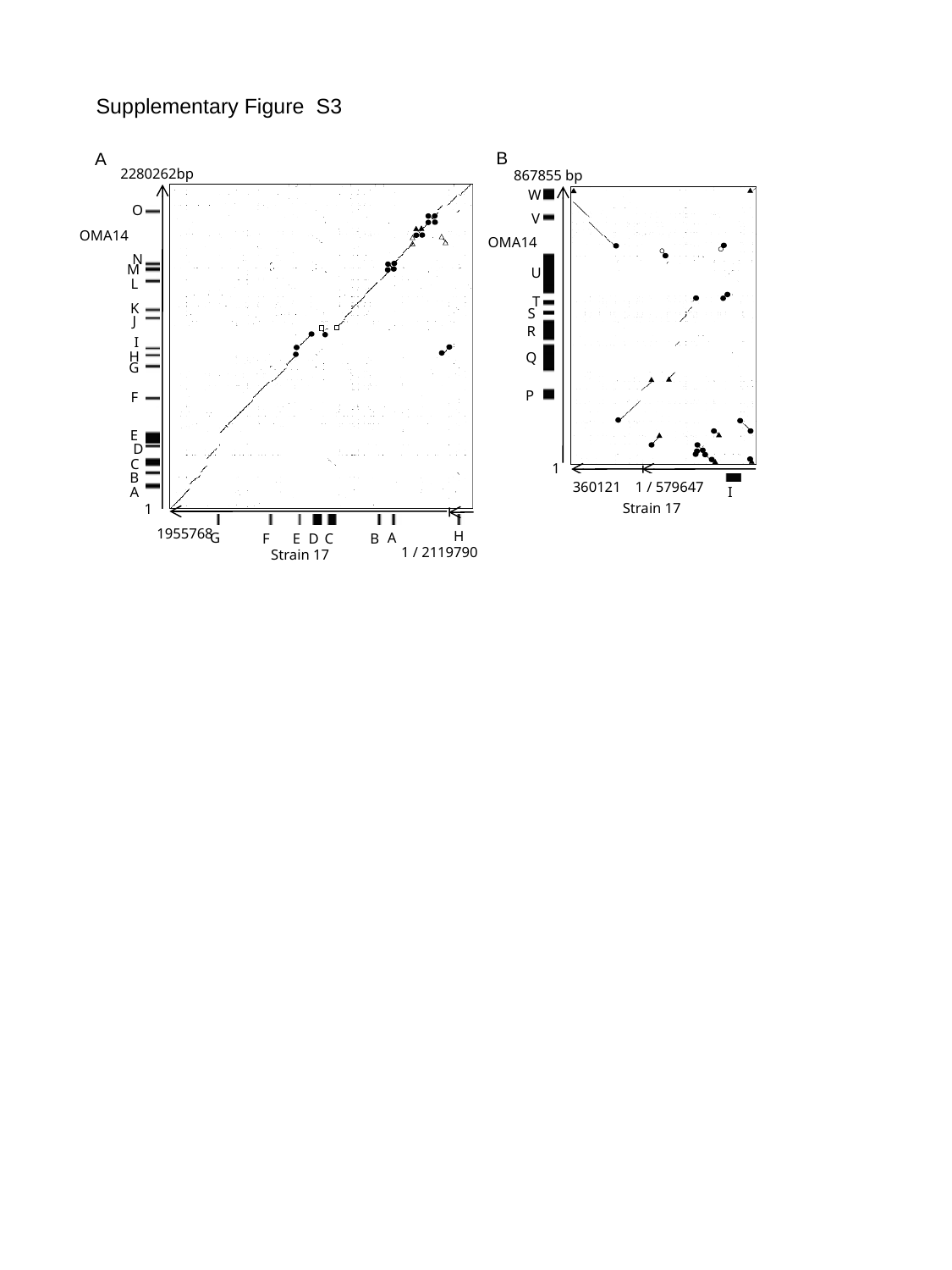

Supplementary Figure S3
B
A
2280262bp
O
OMA14
N
M
L
K
J
I
H
G
F
E
D
C
B
A
1
1955768
H
A
G
F
E
D
C
B
1 / 2119790
Strain 17
867855 bp
W
V
OMA14
U
T
S
R
Q
P
1
360121 1 / 579647
I
Strain 17
