# Supplementary material for: The complete genome sequencing of Prevotella intermedia strain OMA14 and a subsequent fine-scale, intra-species genomic comparison reveal an unusual amplification of conjugative and mobile transposons and identify a novel Prevotella-lineage-specific repeat
Source: DNA Res. 2015 Dec 8;23(1):11–9. doi: 10.1093/dnares/dsv032 (PMC4755523; doi:10.1093/dnares/dsv032)
Supplement: Supplementary Data [file supp_dsv032_dsv032supp_fig4.ppt]

## Slide 1
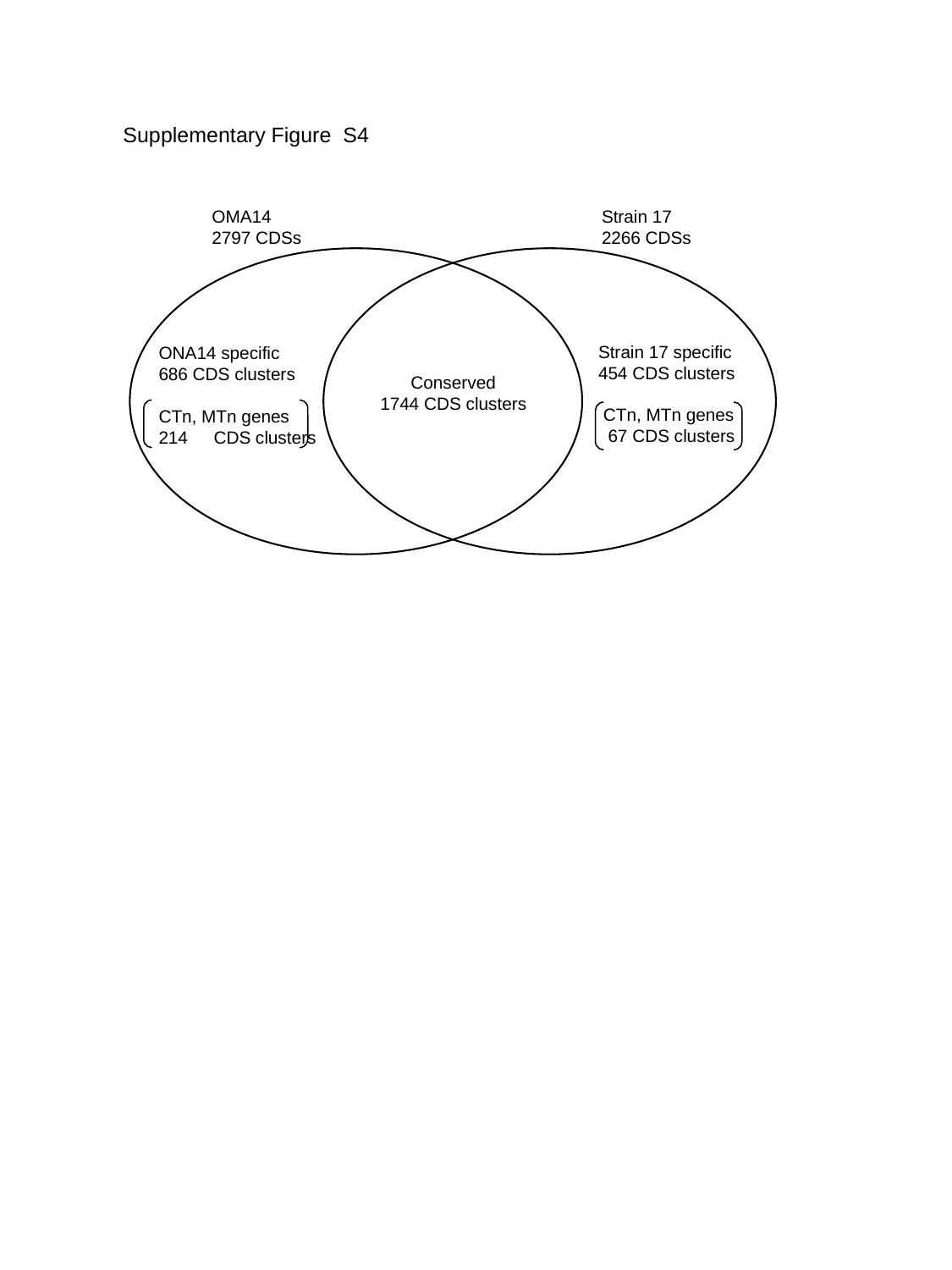

Supplementary Figure S4
OMA14
2797 CDSs
Strain 17
2266 CDSs
Strain 17 specific
454 CDS clusters
 CTn, MTn genes
 67 CDS clusters
ONA14 specific
686 CDS clusters
CTn, MTn genes
214　CDS clusters
Conserved
1744 CDS clusters
